# Chemical Characterization Analysis, Antioxidants, and Anti-Diabetic Activity of Two Novel Acidic Water-Soluble Polysaccharides Isolated from Baobab Fruits

**DOI:** 10.3390/foods13060912

**Published:** 2024-03-17

**Authors:** Shen Song, Mohamed Aamer Abubaker, Maryam Akhtar, Abdalla Musa Elimam, Xinliang Zhu, Ji Zhang

**Affiliations:** 1 College of Life Science, Northwest Normal University, Lanzhou 730070, China; xbsdsongshen@nwnu.edu.cn (S.S.); moaamer2017@gamil.com (M.A.A.); moaamer1990@nwnu.edu.cn (M.A.); widadmoh1990@gmail.com (X.Z.); 2 New Rural Development Research Institute of Northwest Normal University, Lanzhou 730070, China; 3 Department of Biology, Faculty of Education, University of Khartoum, Khartoum 11111, Sudan; 4 Department of Biology, Faculty of Science, Al-Baha University, Al Baha 75311, Saudi Arabia; wadelimam_71@hotmail.com

**Keywords:** *Adansonia digitata*, acidic polysaccharides, antioxidants, anti-diabetic

## Abstract

This study explores the isolation and characterization of two acidic polysaccharides from baobab (*Adansonia digitata*) fruits, named ADPs40-F3 and ADPs60-F3; the two types of acidic polysaccharides exhibited high sugar content and chemical structural features characterized by O–H, C–H, carbonyl C=O, and COOH carboxyl functional groups. The two fractions showed molecular weights of 1.66 × 10^5^ and 9.59 × 10^4^ Da. ADPs40-F3 residues consist of arabinose (2.80%), galactose (0.91%), glucose (3.60%), xylose (34.70%), and galacturonic acid (58.10%). On the other hand, ADPs60-F3 is composed of rhamnose (1.50%), arabinose (5.50%), galactose (2.50%), glucose (3.10%), xylose (26.00%), and galacturonic acid (61.40%). Furthermore, NMR analysis showed that the main acidic structures of ADPs40-F3 and ADPs60-F3 are formed by 4,6)-α-d-Gal*p*A-(1→, →4)-β-d-Xyl*f*-(1→, →4,6)-β-d-Glc*p*-(1→, →5)-α-L-Ara*f*-(1→, →4,6)-α-d-Gal*p*-(1→ residues and 4)-α-d-Gal*p*A-(1→, →4)-β-d-Xyl*f*-(1→, →6)-β-d-Glc*p*-(1→, →5)-α-l-Ara*f-*(1→ 4,6)-α-d-Gal*p*-(4,6→, →2)-α-Rha*p*- residues, respectively, based on the observed signals. Antioxidant assays against DPPH, ABTS^+^, and FRAP revealed significant antioxidant activities for ADPs40-F3 and ADPs60-F3, comparable to ascorbic acid (VC). Additionally, both polysaccharides exhibited a dose-dependent inhibition of α-glucosidase and α-amylase activities, suggesting potential anti-diabetic properties. In vivo evaluation demonstrated that ADPs60-F3 significantly reduced blood glucose levels, indicating promising therapeutic effects. These findings underscore the potential utility of baobab fruit polysaccharides as natural antioxidants and anti-diabetic agents.

## 1. Introduction

To fully understand the relationship between the structure and biological functions of polysaccharides, it is essential to conduct a comparative analysis of their chemical structure, chain conformation, and bioactivities. This knowledge can significantly enhance the applications of natural polysaccharides in biomedical fields [1]. One of the important criteria for classifying natural polysaccharides is their charge property, which depends on the presence or absence of acidic or basic groups in their molecular structure [2]. Ionic polysaccharides can be either anionic (e.g., pectin, alginic acid, alginate, carboxymethyl cellulose, hyaluronic acid, heparin, and chondroitin sulfate) or cationic (e.g., alginate, chitosan, and chondroitin sulfate) [3]. Among them, acidic polysaccharides have received special attention for their biological applications, as they have shown higher bioactive potential than neutral polysaccharide fractions [4]. The acidic groups, such as (–COOH), in acidic polysaccharides, enable them to form electrostatic interactions with target biomolecules like proteins and enzymes, thereby modulating their biological functions [5]. For instance, previous studies on acidic polysaccharides isolated from papaya (*Carica papaya*) and shoot polysaccharides of *Bambusa vulgaris* have revealed their antioxidant properties attributed to the presence of uronic acid functional groups, including galacturonic acid and glucuronic acid [6,7].

Acidic polysaccharides usually contain different molar ratios of rhamnose, arabinose, galactose, glucose, and galacturonic acid, with molecular weights ranging from 10^4^ Da to 10^6^ Da. Several studies have reported that the content of galacturonic acid in acidic polysaccharides is positively correlated with their antioxidant activity [8]. In addition, the presence of arabinose, xylose, galacturonic acid, and low molecular weight features has been associated with anti-diabetic activity by interfering with the binding of enzymes and their receptors. Furthermore, a specific type of acidic polysaccharide that consists mainly of galacturonic acid linked by a (1 → 4) bond has been found to exhibit various health benefits, such as antioxidative, anticancer, blood sugar-reducing, cholesterol-lowering, and anxiety-alleviating effects [9]. Therefore, it is essential to isolate novel acidic polysaccharides and characterize their chemical structures [10,11,12]. In various preceding studies, baobab raw materials derived from leaves and fruits have been recognized for their antioxidant properties and α-glucosidase inhibition, albeit without elucidating the active compound or providing detailed explanations for their bioactivity actions [13]. In this study, two acidic polysaccharides, ADPs40-F3 and ADPs60-F3, were isolated from the fruit of *Adansonia digitata* by hot water extraction and gradient ethanol precipitation. The physicochemical properties, molecular weight, monosaccharide composition, and chemical structure of the two acidic polysaccharides were studied. The results showed that *Adansonia digitata* fruit polysaccharide had strong antioxidant capacity and antihyperglycemic activity in vitro and in vivo. This study will promote the further development of *Adansonia digitata* fruit as food and health products.

## 2. Materials and Experimental

### 2.1. Materials

Phenol, sulfuric acid, standard monosaccharides (Fuc, Rha, Ara, Gal, Glc, Xyl, Man, Fru, Rib, GalA, GulA, GalAC, GlcNAc, GlcA, GlcN, and ManA), AL-01, and 3-Methyl-1-phenyl-2-pyrazoline-5-one (PMP) were purchased from Sigma Aldrich. Porcine pancreatic α-amylase (14 U/mg) and α-glucosidase (50 U/mg) were purchased from Aladdin Co., Ltd. (Shanghai, China) and BASF Biotechnology Co., Ltd. (Hefei, China), along with acarbose, starch, and metformin. All other reagents used were analytical grade.

### 2.2. Preparation and Physicochemical Properties of Acidic Polysaccharides

The *Adansonia digitata* fruit polysaccharides were extracted using the hot water extraction method, with a powder-to-solvent ratio of 1:25 mg/mL. Following a 4 h extraction period at 70 °C, the supernatant was centrifuged and then concentrated to 1/4 of the original volume at 60 °C under reduced pressure. Absolute ethanol was subsequently added to achieve alcohol concentrations of 40% (*v*/*v*) and 60% (*v*/*v*), respectively. The resulting polysaccharides were labelled as ADPs40 and ADPs60. Further separation and purification were conducted using DEAE-52 in the presence of NaCl solutions ranging from 0 M to 0.5 M. This process yielded acid fractions denoted as ADPS40-F3 and ADPS60-F3, respectively.

Physicochemical properties are vital for a comprehensive understanding of the characteristics and behavior of ADPs40-F3 and ADPs60-F3. The quantification of total sugar content (*w*/*w*, %) was carried out using the phenol–sulfuric acid method at a wavelength of 490 nm. Additionally, the sulfuric carbazole reaction was used to confirm the existence of uronic acid content (*w*/*w*, %) in the polysaccharides; uronic acid has a condensation reaction with carbazole in the presence of concentrated sulfuric acid, and the protein content (*w*/*w*, %) of the two fractions was identified and analyzed at a wavelength range of 200–400 nm to confirm the removing of pigment and protein at 260 nm and 280 nm [1].

### 2.3. Chemical Characterizations of Acidic Polysaccharides

#### 2.3.1. Determination of Triple Helix Structure

This study aimed to investigate the conformational structure of polysaccharides in solution by analyzing the complexes formed between Congo red and the polysaccharides. The transition from a triple-helical arrangement to a single-stranded conformation was evaluated by measuring the maximum absorption of the Congo red–polysaccharide solutions at varying NaOH concentrations, ranging from 0 M to 0.5 M [14]. The spectral range of 400–800 nm was used to measure the maximum absorption wavelength. To create a control group, distilled water was used instead of the polysaccharide solution and it was mixed with an NaOH and Congo red solution. The visible spectra of this control group were also scanned using the same procedure as the polysaccharide group mixed with NaOH and Congo red.

#### 2.3.2. Determination of Functional Groups

Fourier transform infrared spectroscopy (FTIR-8400S, Shimadzu, Japan) was utilized to analyze the IR spectra of the acidic polysaccharides. The functional groups were identified by mixing the sample with KBr, pressing it into pellets, and analyzing it in the frequency range of 4000–400 cm^−1^ [15].

#### 2.3.3. Determination of Molecular Weight

The molecular weight (Mw) of the polysaccharides obtained in this study was determined using a combination of size-exclusion chromatography, multi-angle laser photometer (MALLS, λ = 690 nm; DAWN EOS, Wyatt Technology Co., Goleta, CA, USA), and an Optilab refractometer (operating at a wavelength of 690 nm), as described by the method of [16]. The acidic polysaccharides were mixed thoroughly with ultrapure water at room temperature and then passed through a 0.45 μm polysaccharide solution at a concentration of 1 mg/mL with minor modifications. To elute the sample, an Ultra-hydrogel TM column (7.8 × 300 mm, Waters, Milford, MA, USA) was used with ultra-pure water as the mobile phase, a flow rate of 1 mL/min, and an injected mass of 50 μL. The refractive index increment (dn/dc) value of 1.3 mL/g was determined and ASTRA software (Wyatt Technologies, Goleta, CA, USA) was used to analyze the data, along with a cellulose filter to obtain the necessary information.

#### 2.3.4. Determination of Monosaccharides’ Compositions

First, 10 mg of the sample was dissolved in 3 M trifluoroacetic acid (TFA) and incubated at 120 °C for 3 h. The resulting solution was dried, dissolved in pure water, and centrifuged before being diluted and analyzed using HPAEC-PAD with a Dionex Carbopac™ PA20 column. The mobile phase elution contained H_2_O, 200 mM NaOH, 50 mM NaOH, and 200 mM NaOAC, and a standard mixture of monosaccharides was used [17].

#### 2.3.5. Determination of Chain Confirmation

To prepare the samples for atomic force microscopy (AFM) imaging, the dried polysaccharide samples were dissolved in deionized water at a concentration of 10 μg/mL. Approximately 5 μL aliquot of the resulting solution was deposited onto a mica plate and allowed to air-dry overnight. The AFM images were acquired using a tapping mode on a Multimode 8 instrument (USA) and captured using Nano Scope software (Digital Instruments, Santa Barbara, CA, USA). [18].

#### 2.3.6. Determination of Surface Morphology

A thin-layer sample granule was mounted on the copper sample holder with double-sided carbon tape and then coated with gold. Scanning electron microscopy (SEM) was conducted with a 15 kV Quanta 2000FEG scanning electron microscope (Chicago, IL, USA).

#### 2.3.7. Determination of Thermal Stability

The thermal decomposition procedure of the polysaccharide fractions samples was monitored by a thermogravimetric analyzer (TA Discovery SDT 650, New Castle, DE, USA). The known weight of each sample was taken using a microbalance (Ohaus China Company, R71MHD3ZH, Shanghai, China), then the determination of thermal stability was performed using the TGA analyzer with the temperature at 25–900 °C, at a rate of 10 °C/min, under a high purity nitrogen flow of 50 mL/min to ensure an inert atmosphere [19].

#### 2.3.8. Confirmation of the Chemical Structure

The lyophilized samples of 50 mg were deuterated by twice lyophilizing with D2O and were subsequently dissolved in 99.9% D_2_O 0.5 mL prior to NMR measurements. The 1D NMR spectra were acquired using a Bruker AVIII-600 NMR spectrometer (Bruker Corporation, Billerica, MA, USA) at 25 °C.

### 2.4. Antioxidants Assays

#### 2.4.1. DDPH Test

The previously described procedure was followed when performing the DPPH radical scavenging activity [20]. A volume of 2 mL of an ethanol-based DPPH solution of 0.2 mmol/L and 2 mL of an aqueous polysaccharide concentration of 0.1 mg/mL, 0.2 mg/mL, 0.3 mg/mL, 0.4 mg/mL, and 0.5 mg/mL were combined, quickly shaken, and allowed to react for 30 min in the dark. The absorbance at 517 nm was measured using an ultraviolet-visible spectrophotometer, and a blank solution of 2 mL of 100% ethanol was added. We generated VC as a positive control at an equal concentration to the polysaccharide solution. The DPPH radical scavenging activity was determined using Equation (1):

(1) $$DPPH\;radical\;scavenging\left(\%\right)=1-\frac{\left(A_{a}-A_{b}\right)}{A_{c}}\times 100\%$$

#### 2.4.2. ABTS^+^ Test

The assessment of antioxidant activity employed ABTS (2,2-azinobis-6-s-3-ethylbenzothiazoline sulfonic acid) radical cation, following the described method. ABTS radical cation was prepared by combining a 7 mM ABTS solution with 2.45 mM potassium persulfate, allowing the reaction to occur for 16 h in the dark at room temperature, with slight adjustments. Before application, the ABTS solution was diluted with ethanol to achieve an absorbance of 0.70 ± 0.05 at 734 nm. Each sample, ranging in concentration from 100 μg/mL to 500 μg/mL, was introduced to 2.0 mL of the ABTS solution with incremental dilutions of 0.1 mg/mL, 0.2 mg/mL, 0.3 mg/mL, 0.4 mg/mL, and 0.5 mg/mL. Following a 6 min incubation at room temperature, the absorbance at 734 nm was promptly measured [21]. The ABTS scavenging effect was determined using Equation (2):

(2) $$ABTS\;radical\;scavenging\left(\%\right)=1-\frac{\left(A_{1}-A_{2}\right)}{A_{0}}\times 100\%$$

where A_1_ is the absorbance of the sample mixed with ABTS radical solution, A_2_ is the absorbance of the sample without ABTS radical solution, and A_0_ is the absorbance of ABTS radical solution without the sample as a blank control.

#### 2.4.3. Ferric-Reducing Antioxidant Power (FRAP) Test

The Benzie and Strain (FRAP) assay method was modified to measure the sample’s total antioxidant potential [22]. The FRAP assay is used to measure the antioxidant activity of a sample by detecting the transfer of electrons from antioxidants to a solution containing Fe(III). This transfer causes a color change that is detected by measuring absorbance at 593 nm. To perform the assay, a FRAP reagent is created by mixing acetate buffer, TPTZ, and FeCl_3_. The sample is mixed with the FRAP reagent and deionized water, and the mixture is examined three times, with a reading taken 8 min after the sample is added. The FRAP value is calculated by subtracting the initial blank reading from the final reading with the sample. The results are expressed as an antioxidant concentration equivalent to 1 mol/L FeSO_4_–7H_2_O, using FeSO_4_–7H_2_O as a standard curve at various concentrations.

### 2.5. In Vitro and In Vivo Anti-Diabetic Assay

#### 2.5.1. In Vitro Anti-Diabetic Assay

The in vitro hypoglycemic effect of the acidic polysaccharides were investigated through the α-amylase and α-glucosidase inhibitory activity assays. The two types of acidic polysaccharides were previously dissolved in distilled water at various concentrations: 1 mg/mL, 2 mg/mL, 3 mg/mL, 4 mg/mL, and 5 mg/mL. Acarbose and distilled water were used as the positive and blank controls, respectively. The absorbance was measured by a UV-1601 Spectrophotometer (Beijing Ruili Analytical Instrument Co., Ltd., Beijing, China).

#### 2.5.2. In Vivo Assays

We assessed the anti-diabetic potential of acidic polysaccharide using a non-diabetic mouse model. A total of 30 non-diabetic female mice were overnight-fasted for 12 h and then categorized into six groups, with each group consisting of five mice (*n* = 5). The mice were orally administered doses of acidic polysaccharide fraction, dissolved in distilled water, in three treatments group—100 mg/kg, 300 mg/kg, and 500 mg/kg—with a dosage of 250 mg/kg. A positive control group received an oral dose of 5 mg/kg of metformin, a well-known anti-diabetic drug, while the control group received distilled water; the model group included non-diabetic mice and was used for comparison with the experimental group. After a 30 min interval following treatment administration, each of the mice was orally given starch at a dose of 2 g/kg of body weight. Blood glucose levels were measured at baseline 0 min, 60 min, and 120 min after starch administration using tail puncture. Data collected were analyzed to assess the effects of the test substance on blood glucose levels compared to the positive control and control groups [23]. The percentage change in blood glucose levels was calculated using Equation (3):

(3) $$Percentage\;Change\left(\%\right)=\frac{\left(Final\;Value-Initial\;Value\right)}{\;Initial\;Value}\times 100\%$$

## 3. Results and Discussion

### 3.1. Preparation of Two Types of Baobab Fruits’ Acidic Polysaccharides and Primary Properties

**Figure 1:**
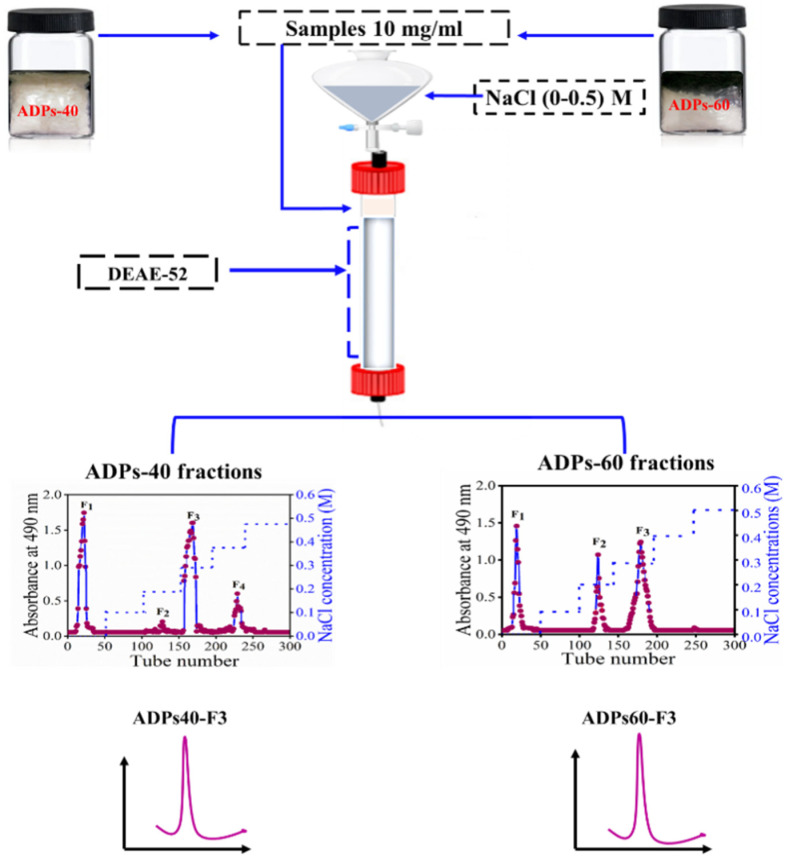
Preparation of the two types of acidic polysaccharides.

After the isolation of *Adansonia digitata* fruits polysaccharides (ADPs), precipitated by absolute ethanol ranging from 40% to 60% led to ADPs-40 and ADPs-60. The two types of acidic baobab fruits’ polysaccharides, ADPs40-F3 and ADPs60-F3, were obtained from the crude polysaccharides ADPs-40 and ADPs-60 after purification and fractionation via DEAE-52 and NaCl solution at different concentrations, as shown in Figure 1.

The two obtained types of acidic polysaccharides ADPs40-F3 and ADPs60-F3 yielded 22.30 ± 0.70% and 23.50 ± 0.60%, respectively. The contents of total sugar were 82.10 ± 1.23% and 85.30 ± 1.21%, uronic acid was 15.00 ± 2.30% and 19.10 ± 1.60%, and protein was 0.38 ± 0.10% and 0.28 ± 0.05%, respectively, with the results shown in Table 1.

**Figure 2:**
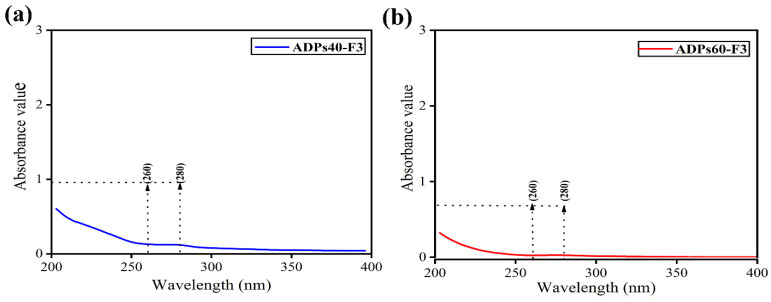
UV-visible absorption of ADPs40-F3 (**a**) and ADPs60-F3 (**b**).

Moreover, UV-visible absorption (Figure 2a,b) reveals that ADPs40-F3 and ADPs60-F3 had no clear absorption signal around 260–280 nm, which confirms that a large amount of protein was removed during the purification process, and the protein content of ADPs40-F3 and ADPs60-F3 was only 0.38 ± 0.10% and 0.28 ± 0.05%, respectively.

### 3.2. Characterization Analysis Results of Acidic Polysaccharides

#### 3.2.1. Triple Helix Structure

Triple helix conformation of polysaccharides is naturally considered to be related to their biological activities [24]. According to the reported literature, polysaccharides with triple-helix could join with Congo red reagent and create a Congo red–polysaccharide complex. The hydrogen connection between the hydroxyl groups in polysaccharides is reduced by a rise in the concentration of NaOH, which results in the loss of the helical shape. In light of this, it is possible to determine the polysaccharide’s helical structure using this feature [25].

**Figure 3:**
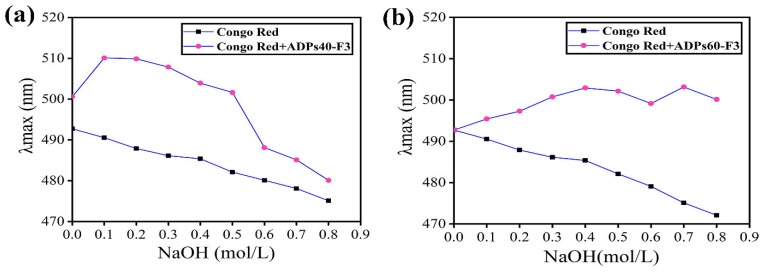
Congo red test of ADPs40-F3 (**a**) and ADPs60-F3 (**b**).

Figure 3 presents the observed maximum absorption wavelengths of both Congo red with fractions and Congo red, which exhibited an increasing and decreasing tendency with the increased concentration of NaOH solutions. The maximum absorption wavelength of the complex in Congo red, with -ADPs40-F3, increased in weakly alkaline solutions from 0.1 mol/L to 0.5 mol/L, as shown in Figure 3a, while it decreased in strongly alkaline solutions at 0.6 mol/L, 0.7 mol/L, and 0.8 mol/L. On the other hand, the maximum absorption wavelengths of Congo red -ADPs60-F3 at different concentrations of NaOH obviously increased from 0.1 mol/L to 0.7 mol/L, as shown in Figure 3b, then decreased at 0.6 mol/L and 0.8 mol/L. These results suggested that the two polysaccharides had triple-helix conformation and were consistent with the results of the AFM study [26].

#### 3.2.2. FTIR Analysis

**Figure 4:**
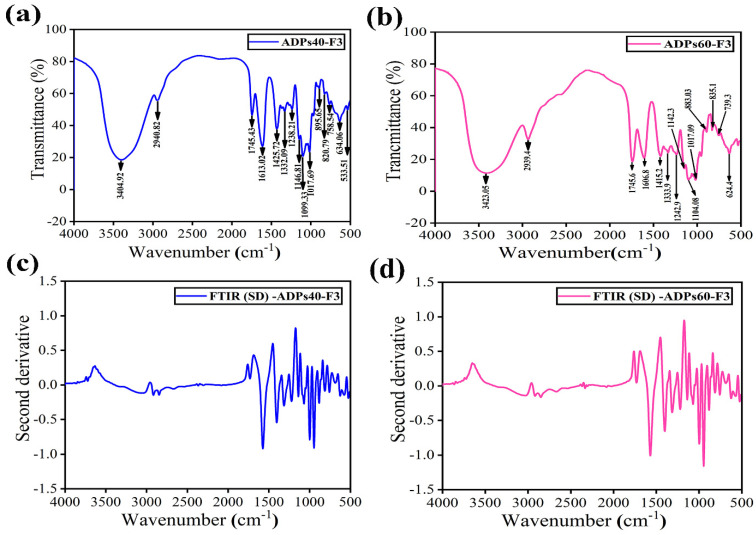
FT-IR and second derivative profile of ADPs40-F3 (**a**,**c**) and ADPs60-F3 (**b**,**d**).

The FTIR spectrum profile of ADPs40-F3 and ADPs60-F3 polysaccharides are shown in Figure 4 and Table 2. ADPs40-F3 showed about 15 absorbance bands and ADPs60-F3 exhibited 14, as presented in Figure 4a,b.

The two types of acidic polysaccharides showed a strong and broad band at 3404.92 cm^−1^ and 3423.05 cm^−1^, which were identified as the peaks of the hydroxyl group –OH stretching vibration peak, and a weak peak attributed to C–H stretching vibration at 2940.82 cm^−1^ and 2939.4 cm^−1^ in two polysaccharides, respectively. The strong and sharp bands found at the 1745.43 cm^−1^ and 1745.6 cm^−1^ regions indicated the carboxylic functional group –COOH and 1608.64 cm^−1^ and 1606.8 cm^−1^ indicated carbonyl C=O of carboxyl [17]. The two medium peaks shown in regions 1425.72 cm^−1^ and 1415.2 cm^−1^ proved the existence connected to the stretching of the methyl ester group in the pectin–CH3. These perhaps indicate the esterification of some of the uronic acid carboxyl [27].

The functional group C–H has appeared in two peaks stretched at the 1332.09 cm^−1^ and 1333.9 cm^−1^ regions, and this agrees with [28]. The peaks appeared at 1146.81 cm^−1^, 1142.3 cm^−1^, 1104.08 cm^−1^, 1098.54 cm^−1^, 1015.25 cm^−1^, and 1017.69 cm^−1^ because of the stretching vibrations of C=O, C–O–C glycosidic, which are outcomes from the existence of pyranose-form sugar, such as glucose [29]. The absorbance bands area in 800–1200 cm^−1^ was identified by carbohydrate fingerprint [30]. The three appeared peaks after 800 cm^−1^ were assigned to the β-glycosidic and α-glycosidic bonds configuration [31]. The FT-IR spectrum second derivative, shown in Figure 4c,d, was used to increase the resolution of the one-dimensional infrared spectrogram [32]. The FTIR results of the fractions agreed with this study, with slight difference as acidic polysaccharides [33].

#### 3.2.3. Molecular Weight Analysis

**Figure 5:**
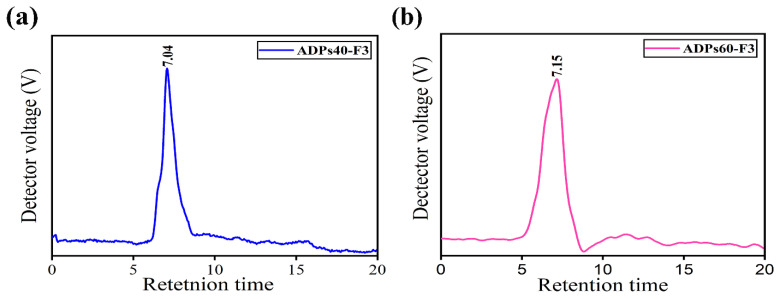
Molecular weight profiles of ADPs40-F3 (**a**) and ADPs60-F3 (**b**).

The polysaccharides’ molecular weights reflect polysaccharide chains and are neatly correlated with the biological activities of polysaccharides [34]. The molecular weights of ADPs40-F3 and ADPs60-F3 polysaccharides evaluated by gel permeation chromatography using dextran standards are shown in Figure 5a,b. The molecular weights of ADPs40-F3 and ADPs60-F3 were 1.66 × 10^5^ Da and 9.59 × 10^4^ Da, respectively.

The ADPs40-F3 and ADPs60-F3 homogeneity was estimated by a polydispersity index (PDI) of 1.85 Da and 1.63 Da, respectively. This indicates that the ADPs60-F3 polysaccharide shows a higher homogeneity than ADPs40-F3, and both ADPs40-F3 and ADPs60-F3 had single peaks in the chromatogram. The molecular weight corresponded to the molecular distribution, as shown in Table 3.

#### 3.2.4. Monosaccharides’ Compositions Analysis

**Figure 6:**
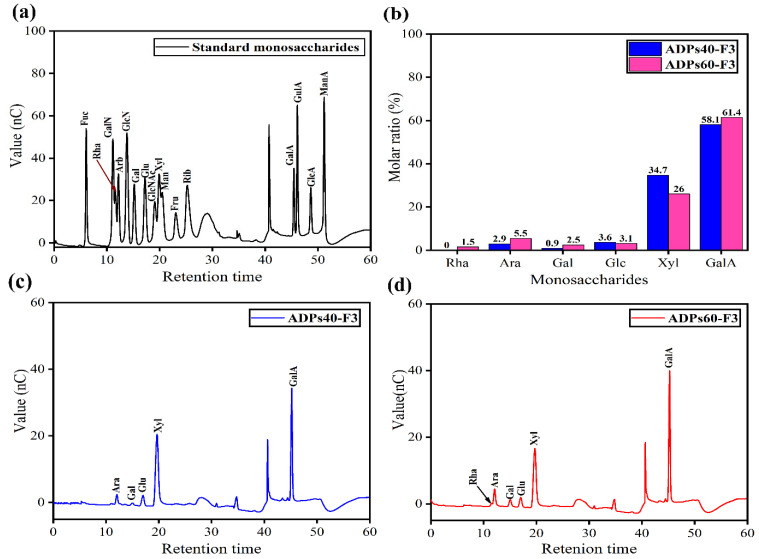
Standard residues (**a**), molar ratio % (**b**), ADPs40-F3 residues (**c**), and ADPs60-F3 residues (**d**).

The monosaccharide composition of ADPs40-F3 and ADPs60-F3 polysaccharides was measured via HPAEC-PAD analysis after TFA hydrolysis. The samples were identified by matching their retention time with those of standard monosaccharides in Figure 6a under the same analytical conditions.

Monosaccharide composition analysis indicated that ADPs40-F3 are composed of five monosaccharides residues, as shown in Figure 6c, namely, arabinose (Ara), galactose (Gal), glucose (Glc), xylose (Xyl), and galacturonic acid (GalA), with molar ratio %s of 2.80:0.91:3.60:34.70:58.10, respectively. APDs60-F3 polysaccharide made up six monosaccharides residues, as shown in Figure 6d, namely, rhamnose (Rha), arabinose (Ara), galactose (Gal), glucose (Glc), xylose (Xyl), and galacturonic acid (GalA), with molar ratio %s of 1.50:5.50:2.50:3.10:26.00:61.40, respectively. The major monosaccharides composition in ADPs40-F3 and ADPs60-F3 are Glc, Xyl, and GalA. The Rha monosaccharide exists in polysaccharide ADPs60-F3; in contrast, it disappears in polysaccharide ADPs40-F3 because of the obvious difference in the monosaccharides’ composition units that make up these polysaccharides classified as hetero-polysaccharides. The most abundant monosaccharide in two polysaccharides was galacturonic acid (GalA), with a molar ratio of 58.10% and 61.40%, respectively, as shown in Table 4. With the presence of galacturonic acid (GalA) in this amount in both polysaccharides, it will obviously lead to classifying these two polysaccharides as acidic polysaccharides, and the results agreed with [35].

#### 3.2.5. AFM Analysis

**Figure 7:**
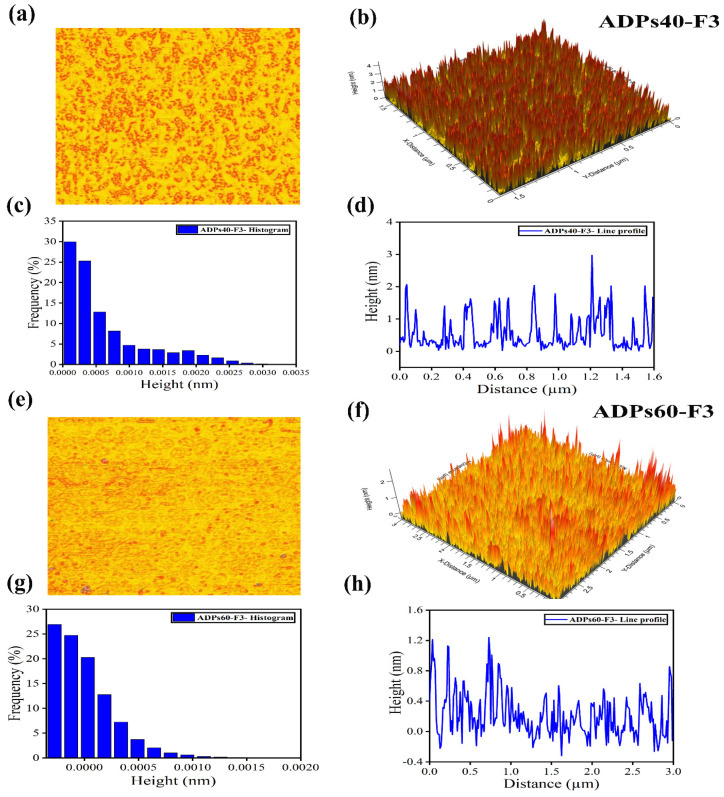
AFM profile of ADPs40−F3 (**a**–**d**) and ADPs60−F3 (**e**–**h**).

AFM analysis can provide valuable insights into the branching patterns and mechanical properties of polysaccharides, making it a useful tool for the characterization of these complex biomolecules, and for describing the protuberances and aggregations of ADPs40-F3 and ADPs60-F3, as shown in Figure 7. The two polysaccharides are made up of numerous protuberances with an obvious length and are not uniform. The AFM results include two-dimensional (2D) and three-dimensional (3D), whereas it describes the distribution in the two polysaccharides as uniform and with a different size and height. The average surface roughness (ASR) was 4 nm and 2 nm, and this was because of the low molecular weight compared to the previous one. Added to that, the protuberances on polysaccharides can reveal essential details about their function and characteristics depending on their size, shape, and distribution. Protuberances, for instance, can be involved in the adherence of bacterial polysaccharides to host tissues or other surfaces as well as the development of biofilms. In the field of food science, polysaccharides’ texture and mouthfeel can be impacted by protuberances. The hydroxyl groups in the polysaccharides’ skeletal form provide hydrogen bonds to form the aggregation of the fractions; this is due to high intermolecular and intramolecular interaction between the residues [36].

Overall, acidic polysaccharide fractions can be subjected to AFM analysis to learn more about their physical characteristics and behavior. This information can be helpful in a variety of sectors, such as biotechnology, materials science, and food science.

#### 3.2.6. SEM Analysis

**Figure 8:**
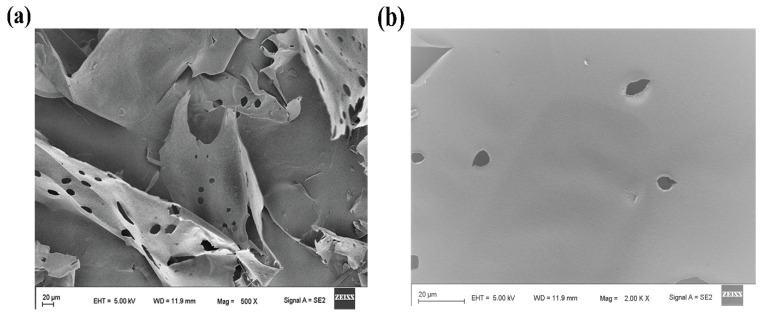
Surface morphological of ADPs40-F3 (**a**) and ADPs60-F3 (**b**).

Here, we study the modification in the surface morphological of the fractions ADPs40-F3 and ADPs60-F3, as shown in Figure 8.

After purification and fractionation, we can see the two types of acidic polysaccharides, ADPs40-F3 in Figure 8a and ADPs60-F3 in Figure 8b; the shape is unified compared to the polysaccharide before the purification and separation process, which appears with a thin slice shape in the honeycomb structure [37]. It shows a smooth and thin layer surface, which agrees with the results of the polysaccharides obtained from *Moringa oleifera Lam*. leaves [38].

#### 3.2.7. TGA Analysis

**Figure 9:**
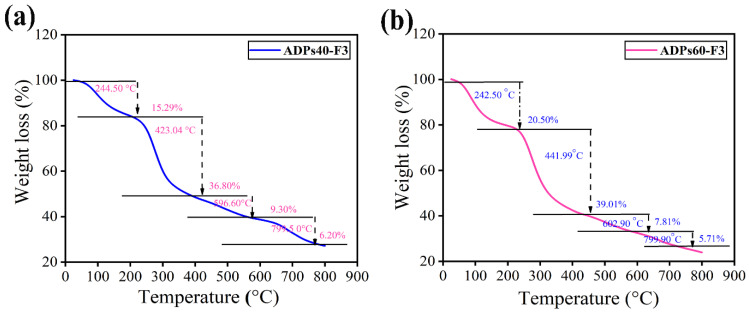
TGA curves of ADPs40-F3 (**a**) and ADPs60-F3 (**b**).

In Figure 9, the thermogravimetric analysis (TGA) curves of polysaccharides ADPs40-F3 and ADPs60-F3 at the two initial stages (*Ti*) 242.50 °C, and 244.50 °C indicate low weights of 15.29% and 20.50%, respectively. This decrease was caused by moisture as hydrogen-bonded water departed the polysaccharide structure [39]. With weight losses of 36.80% and 39.01%, respectively, the TGA of the two types of polysaccharides at the second stage similarly exhibited a sharp weight loss stage at 423.04 °C in polysaccharide ADPs40-F3 and 441.99 °C in ADPs60-F3. This could be because of polysaccharide heat degradation and biopolymer structural collapse.

The half-life temperature (*T50*) indicates the temperature at which the mass loss ratio of the sample was 50%. The *T50* of ADPs40-F3 and ADPs60-F3 were 423.04 °C and 441.99 °C, respectively. Because of exciting functional groups in both fractions, therefore, the two fractions show thermal stability [40], and from the TGA results, we can say that the two types of acidic polysaccharides have strong thermal stability, as shown in Table 5.

The TGA curve can reveal important details about the thermal stability of polysaccharides, such as when degradation begins and ends, what temperature decomposition occurs at, and how much residual mass remains after decomposition. With this knowledge, processing conditions for polysaccharide-based goods like food ingredients, medication delivery systems, or biopolymer films can be improved.

#### 3.2.8. NMR Analysis

**Figure 10:**
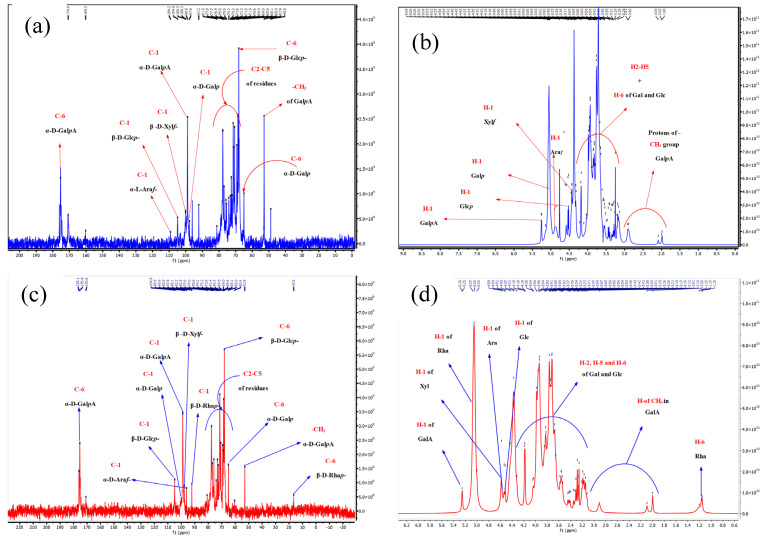
One-dimensional and two-dimensional NMR of ADPs40-F3 (**a**,**c**) and ADPs60-F3 (**b**,**d**).

The 1D/NMR spectra was employed for further identification of the ADPs40F-3 and ADPs60-F3 chemical structure, as shown in Figure 10. The ^13^C-1H NMR signals showed polysaccharides characterize regions like δC 60–110 ppm and δH 3.00–5.50 ppm [41], which elucidated that the two acidic polysaccharides have the two types of α and β, and linkages confirmed the pyranose and furan rings of the monosaccharides’ residuals. Generally, the chemical shifts of anomeric in 13C-1H NMR at 100 ppm and >4.90 ppm, respectively, indicated that α-glycosidic and β-glycosidic are present in the FTIR wavenumber. ADPs40-F3 and ADPs60-F3 showed a quaternary carbon signal at δC 176.1 ppm, and δC175.4 ppm indicated the presence of C-6 of -α-Gal*p*A- of galacturonic acid (GalA), the main residue in the acidic polysaccharides. Added to that, the existence of δC 160.3 ppm and δC 170.8 ppm in the two acidic polysaccharides, respectively, indicated branched and unbranched residues, which agrees with the AFM results as a technique of confirmation aggregation [42]. In the 13C NMR spectra of ADPs40-F3 and ADPs60-F3, distinct signals were observed in the δC 60–80 ppm range, corresponding to the presence of carbon atoms C2-C5 in the polysaccharide residues. In ADPs40-F3, specific chemical shifts observed were δC 81.3, 77.8, 77.7, 76.9, 75.8, 75.5, 74.1, 73.2, 72.6, 71.4, 71.3, 70.6, 70.4, 69.0, 68.3, 68.1, and 52.8 ppm. Among these, the signals at δC 68.9 ppm and δC 65.0 ppm were attributed to the carbon atoms C-6 of β-D-Glc*p* and α-D-Gal*p*, respectively. In contrast, in ADPs60-F3, the observed chemical shifts were δC 80.9, 77.7, 77.6, 77.0, 75.8, 73.2, 72.6, 71.4, 70.7, 69.3, 69.0, 68.1, and 65.0 ppm. Among these, the signals at δC 69.0 ppm, δC 65.0 ppm, and δC 16.0 ppm were assigned to the carbon atoms C-6 of β-d-Glc*p*, α-d-Gal*p*, and α-Rha*p*, respectively. The signal that appeared at δC 65–80 ppm in the 13C NMR of the two fractions exhibited the presence of pyranose residues units like β-d-Glc, α-d-Gal, α-d-Xyl, α-d-GalA, and α-d-Rha and furanose like α-d-Ara [43]. The 13C NMR of ADPs60-F3 in Figure 9c showed signals with high and clear intensity at signal δC 16.5 ppm; this signal is attributed to the methyl carbon (–CH3) of rhamnose (Rha) residue. On the other hand, the signal disappeared in 13C NMR of ADPs40-F3. Added to that, the 13C NMR of ADPs60-F3 signals δC 92.0 ppm were identified to be the C-1 of α-Rha (→2)-*α*-Rha*p*, respectively, which also disappeared in the 13C NMR of ADPs40-F3, with the results agreeing with monosaccharides’ compositions analysis [44].

Corresponding to the 1H NMR results, the ADPs40-F3 fraction showed several anomeric signals at δH 5.25, 5.02, 5.09, 5.00, 4.76, and 4.52 ppm. ADPs60-F3 revealed anomeric region signals at δH 5.25, 5.07, 5.02, 4.54, and 4.54 ppm, which confirmed α- and β-type configuration of sugars; there was a clear intensity in the 1H NMR of the two fractions at δH 4.70 ppm indicated to D_2_O [45]. This conclusion further confirmed the results of monosaccharide compositional analysis, as shown in Table 6.

**Figure 11:**
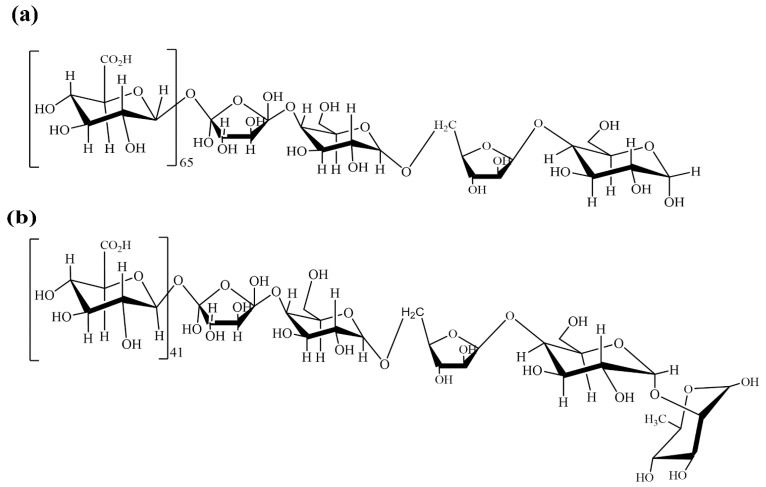
Chemical structure of ADPs40-F3 (**a**) and ADPs60-F3 (**b**).

The ^13^C-^1^H nuclear magnetic resonance (NMR) spectra provided valuable insights into the structural composition of the polysaccharides. In the case of ADPs40-F3, specific chemical shifts observed in the NMR spectra were assigned to the presence of 4,6)-α-d-Gal*p*A-(1→, → 4)-β-d-Xyl*f*-(1→, →4,6)-β-d-Glc*p*-(1→, →5)-α-l-Ara*f*-(1→, →4,6)-α-d-Gal*p*-(1→ residues. Similarly, the 13C-1H NMR spectra of ADPs60-F3 indicated the presence of 4)-α-d-Gal*p*A-(1→, →4)-β-d-Xylf-(1→, →6)-β-d-Glc*p*-(1→, →5)-α-l-Ara*f*-(1→ 4,6)-α-d-Gal*p*-(4,6→, →2)-*α*-Rha*p-* residues based on the observed signals. By considering the molar ratio analysis of the two acidic polysaccharides, we can estimate the number of repeat units present in the ADPs40-F3 fraction, approximately, every 65 units of D-GalpA, 38 units of D-Xyl*f,* 4 units of D-Glc*p*, 3 units of D-Ara*f*, and 1 unit of D-Gal*p*, that are expected to be present. Similarly, in the ADPs60-F3 fraction, the estimated composition suggests approximately 41 units of D-Gal*p*A, 17 units of D-Xyl*f*, 2 units of D-Glc*p*, 2 units of D-Gal*p*, 4 units of D-Ara*f*, and 1 unit of α-D-Rha*p*-, as repeat units analyzing the molar ratio allows us to gain insights into the repeating patterns and relative abundance of different monosaccharide residues in the polysaccharide structure, as shown in Figure 11.

### 3.3. Antioxidant Analysis

**Figure 12:**
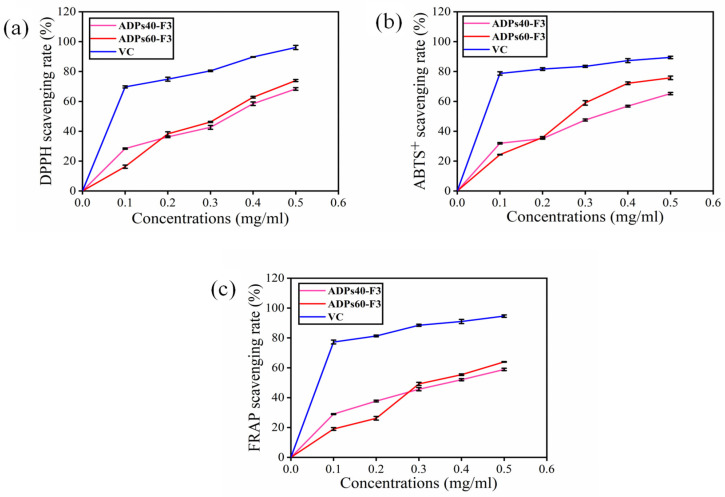
Acidic polysaccharides ADPs40-F3 and ADPs60-F3 antioxidant activity DPPH (**a**), ABTS^+^ (**b**), and FRAP (**c**), with the data expressed using mean ± SD.

In this study, several in vitro antioxidant tests were performed to evaluate the baobab polysaccharides fractions, which include DPPH radical, ABTS, and FRAP assays. The DPPH technique is a widely used method to measure the ability of natural substances to scavenge free radicals. These tests help to assess the potential of baobab polysaccharides fractions as antioxidants [46]. Therefore, a substance’s capacity to scavenge the DPPH free radical is used to describe its antioxidant activity. In Figure 12, this experiment demonstrates the two pure types of acidic polysaccharides’ capacity against scavenge DPPH, ABTS^+^, and FRAP free radicals, with the findings showing that the two types of acidic polysaccharides with VC as control produced significant antioxidant activity at different concentrations. As shown in Figure 12a, against DPPH with a dose of 0.1 mg/mL, the VC inhibition rate was 69.70 ± 0.72%, ADPs40-F3 was 28.00 ± 0.40%, and ADPs60-F3 was 16.21 ± 1.11%. With a dose of 0.5 mg/mL, VC showed an inhibition rate of 96.11 ± 1.43%, while ADPs40-F3 and ADPs60-F3 displayed rates of 68.32 ± 0.92% and 73.90 ± 0.73%, respectively.

In Figure 12b, the ABTS^+^ with a dose of 0.1 mg/mL, the VC inhibition rate was 78.70 ± 1.15%, ADPs40-F3 was 31.90 ± 0.52%, and ADPs60-F3 was 24.30 ± 0.26%. With a dose of 0.5 mg/mL, VC showed an inhibition rate of 89.41 ± 0.88%, while ADPs40-F3 and ADPs60-F3 exhibited rates of 65.21 ± 0.55% and 75.73 ±1.21%, respectively. In Figure 12c, the FRAP with a dose of 0.1 mg/mL, the VC inhibition rate was 77.22 ± 1.35%, ADPs40-F3 was 28.9 ± 0.34%, and ADPs60-F3 was 19.01 ± 0.93%. With a dose of 0.5 mg/mL, VC showed an inhibition rate of 94.72 ± 0.83%, while ADPs40-F3 and ADPs60-F3 revealed rates of 58.80 ± 0.84% and 63.41 ± 0.25%, respectively. The two types of acidic polysaccharides demonstrated significant potency in terms of antioxidants, and this might be due to the monosaccharides and functional groups: precisely, –COOH derived from galacturonic acid (GalA).

### 3.4. Anti-Diabetic Analysis

#### 3.4.1. α-Amylase and α-Glucosidase Inhibitory Activity Assays

**Figure 13:**
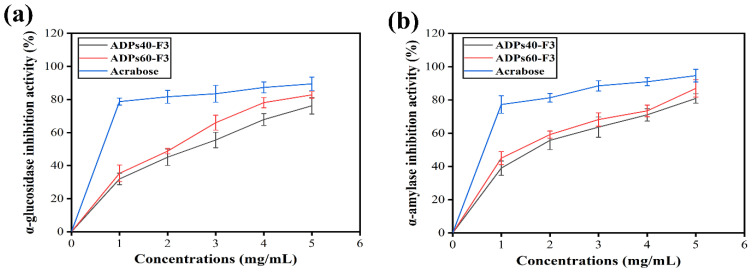
Inhibition effects of ADPs40-F3 and ADPs60-F3 on α-glucosidase (**a**) and α-amylase (**b**), with the data expressed using mean ± SD.

The α-glucosidase and α-amylase inhibitory activity assays are widely used to determine the in vitro hypoglycemic effects of bioactive compounds from natural sources, precisely, polysaccharides [47]. Certain polysaccharides possess the ability to modulate the activity of enzymes and hormones involved in glucose metabolism. For instance, polysaccharides derived from medicinal plants have been shown to inhibit the activity of enzymes like α-amylase and α-glucosidase, which are involved in carbohydrate breakdown. By inhibiting these enzymes, the rate of glucose release from complex carbohydrates is reduced, leading to improved blood sugar control. Moreover, this study has proved that the two types of acidic polysaccharides showed excellent α-glucosidase and α-amylase inhibitory activities. As shown in Figure 13, acidic polysaccharides derived from baobab fruits exhibited concentration-dependent inhibitory effects on α-glucosidase and α-amylase in a dose-dependent manner at 1 mg/mL, 2 mg/mL, 3 mg/mL, 4 mg/mL, and 5 mg/mL. The inhibition rates of ADPs40-F3, ADPs60-F3, and acarbose at the highest dose of 5 mg/mL on the activities of α-glucosidase, as shown in Figure 12a, were 76.22 ± 5.02%, 82.78 ± 2.02%, and 89.44 ± 4.08%, respectively.

On the other hand, the two types of acidic polysaccharides and acarbose also showed an inhibition effect against α-amylase, as shown in Figure 13b, in which the inhibition rates of 5 mg/mL were 80.87 ± 2.80%, 86.94 ± 5.20%, and 94.61 ± 3.50%, respectively. As we can see, acarbose indicated a higher inhibitory effect than the two types of polysaccharides, due to acarbose having a lower molecular weight compared to the two types of acidic polysaccharides in conducive-to-emerging enzyme-inhibitor complexes [48]. In contrast, because of the presence of hydroxyl and ketone groups, specifically the carboxyl group (–COOH), in two types of acidic polysaccharides, it led to increases in their affinity for enzymes, forming complexes. Within these complexes, the polysaccharides capture hydrogen ions released from the enzymes’ catalytic sites, causing alterations in polarity and molecular conformation; consequently, this leads to a partial inhibition of enzyme activity [49]. Therefore, the observed inhibitory effects of the two types of acidic polysaccharides on α-amylase and α-glucosidase can be attributed to factors such as the molecular weight, as well as the presence of uronic acid composition and arabinose and xylose residues. The ADPs60-F3, in contrast to ADPs40-F3, demonstrated superior inhibitory effects against both enzyme types. Consequently, ADPs60-F3 was chosen for subsequent in vivo investigations.

#### 3.4.2. In Vivo Analysis

In vivo analysis results of the blood glucose level measurements reveal significant insights into the anti-diabetic potential of the tested treatments, as shown in Table 7. In the control group, there were only marginal fluctuations in blood glucose levels over the 120 min period, indicating the stability of the experimental conditions, with an average change of less than 2%. In contrast, the model group exhibited a substantial increase in blood glucose levels, with a remarkable rise from the baseline, by approximately 191% at the 60 min interval and 197% at the 120 min interval, clearly showcasing the successful induction of the diabetic condition in these mice. Notably, the metformin-treated group displayed a substantial reduction in blood glucose levels, with an impressive decrease of approximately 41% at the 60 min interval and 45% at the 120 min interval, showcasing the effectiveness of metformin as a standard anti-diabetic drug. Importantly, the groups treated with polysaccharides at different doses exhibited intriguing trends. The low-dose polysaccharide group demonstrated a consistent decline in blood glucose levels, with a notable reduction of approximately 23% at the 60 min interval and 15% at the 120 min interval, although this was not as pronounced as the metformin group. The medium-dose polysaccharide group exhibited a similar pattern, with a decrease of approximately 33% at the 60 min interval and 22% at the 120 min interval. Strikingly, the high-dose polysaccharide group displayed a remarkable reduction in blood glucose levels, which was nearly comparable to the metformin-treated group, with a significant decrease of approximately 44% at the 60 min interval and 29% at the 120 min interval. These findings suggest that the ADPs60-F3 polysaccharides, particularly at higher doses, possess substantial anti-diabetic properties, potentially offering a natural and effective alternative or supplement to conventional anti-diabetic medications.

## 4. Conclusions

This study aimed to isolate two types of acidic polysaccharides, identified as ADPs40-F3 and ADPs60-F3; these polysaccharides exhibited high sugar content and demonstrated structural characteristics indicative of potential therapeutic benefits. Both ADPs40-F3 and ADPs60-F3 displayed significant antioxidant activity against DPPH and ABTS^+^ radicals, suggesting their potential as natural antioxidants. Furthermore, the polysaccharides exhibited a dose-dependent inhibition of α-glucosidase and α-amylase activities, highlighting their promising antihyperglycemic properties.

In the in vivo evaluation, ADPs60-F3 showed a notable reduction in blood glucose levels, which was comparable to the effects observed with metformin treatment. These findings underscore the potential of ADPs40-F3 and ADPs60-F3 as natural agents for managing diabetes and oxidative stress-related disorders. Further research is warranted to elucidate the underlying mechanisms of action and to assess the clinical efficacy of these acidic polysaccharides derived from *Adansonia digitata* fruits. This investigation establishes a solid theoretical and experimental foundation for the utilization of baobab fruit and its polysaccharides, inspiring the development of innovative functional foods and pharmaceuticals.

## Figures and Tables

**Table 1:** Physicochemical properties of ADPs40-F3 and ADPs60-F3.

Features	ADPs40-F3	ADPs60-F3
Yield amounts (*w*/*w*,%)	22.30 ± 0.70	23.50 ± 0.60
Total sugar contents (*w*/*w*,%)	82.10 ± 1.23	85.30 ± 1.21
Uronic acid (*w*/*w*,%)	15.00 ± 2.30	19.10 ± 1.60
Protein content (*w*/*w*,%)	0.38 ± 0.10	0.28 ± 0.05
Molecular weight (Da)	1.66 × 10^5^	9.59 × 10^4^
Water solubility	soluble	soluble

**Table 2:** FT-IR spectrum profile of ADPs40-F3 and ADPs60-F3.

Primary Type of Vibrations	Frequency (cm^−1^) Primary		
	ADPs40-F3	ADPs60-F3	Relative Intensities
v (–OH)	3404.92	3423.05	(br)
v (C–H)	2940.82	2939.40	(w)
v (COOH)	1745.43	1745.60	(s)
v (C–O)	1613.02	1606.80	(s)
v (C=O)	1425.72	1415.20	(m)
v (C–O)	1332.09	1333.90	(m)
v (C–O–C)	1146.81	1242.90	(m)
	1094.33	1142.30	(m)
	1015.25	1104.08	(w)
β-configuration	895.65	1017.09	(w)
	820.79	883.03	(w)
	738.54	835.10	(w)
α-configuration	634.06	739.30	(w)
	533.51	624.40	(m)

Notes: v = vibration; br = broad; w = weak; s = strong or sharp; m = medium.

**Table 3:** Molecular weights of ADPs40-F3 and ADPs60-F3.

Sample	MW (Da)	Mn (Da)	Mw/Mn (Da)
ADPs40-F3	1.66 × 10^5^	8.96 × 10^4^	1.85
ADPs60-F3	9.59 × 10^4^	5.63 × 10^4^	1.63

**Table 4:** Monosaccharides composition of ADPs40-F3 and ADPs60-F3.

Polysaccharides	Residues	R.T. (min)	Molar Ratio (%)
ADPs40-F3	Arabinose	12.00	2.80
	Galactose	14.91	0.91
	Glucose	17.00	3.60
	Xylose	19.62	34.70
	Galacturonic acid	45.21	58.10
ADPs60-F3	Rhamnose	11.50	1.50
	Arabinose	12.00	5.50
	Galactose	15.00	2.50
	Glucose	17.00	3.10
	Xylose	19.70	26.00
	Galacturonic acid	45.20	61.40

**Table 5:** TGA analysis of ADPs40-F3 and ADPs60-F3.

Fractions	Temperature (°C)	Weight Loss (%)	(Ti)	(T50)
ADPs40-F3	244.50	15.29	(Ti)	
	423.04	36.81		(T50)
	596.60	9.30		
	799.50	6.20		
ADPs60-F3	242.50	20.50	(Ti)	
	441.99	39.01		(T50)
	602.90	7.81		
	799.90	5.71		

**Table 6:** 1H and 13C NMR Chemical Shifts for ADPs40-F3 and ADPs60-F3.

Fractions	Residues	Chemical Shifts, δC-δH ppm					
		C1 H1	C2 H2	C3 H3	C4 H4	C5 H5	C6 H6
ADPs40-F3	4,6)-α-d-Gal*p*A-(1→	98.9 4.91	68.1 3.67	68.6 3.96	77.7 4.43	70.4 5.09/5.02	170.7 Nd
	4)-β-d-Xyl*f*-(1→	100.2 4.45	74.1 3.60	72.6 3.71	75.8 3.52	68.1 4.13	Nd Nd
	6)-β-d-Glc*p*-(1→	104.9 4.52	73.2 3.33	76.9 3.43	71.3 3.40	75.5 3.65	68.9 3.85/4.32
	5)-α-l-Ara*f*-(1→	109.2 4.54	81.3 4.03	76.9 3.91	77.8 3.58	52.8 4.04	Nd Nd
	4,6)-α-d-Gal*p*-(1→	97.8 5.02	69.2 3.85	71.4 3.89	70.6 4.18	68.3 4.19	65.0 3.91a/3.67b
ADPs60-F3	4,6)-α-d-Gal*p*A-(1→	98.9 5.26	68.1 3.67	68.6 3.96	77.6 4.43	70.7 5.07/-	170.7 Nd
	4)-α-d-Xyl*f*-(1→	97.9 4.58	71.4 3.29	75.8 3.91	70.7 3.77	65.0 3.41	Nd Nd
	6)-β-d-Glc*p*-(1→	104.9 4.52	73.2 3.33	77.0 3.43	71.4 3.41	75.8 3.65	69.0 3.85a/4.32 b
	5)-α-d-Ara*f*-(1→	96.1 4.54	80.9 4.03	75.8 3.91	77.6 3.58	68.6 4.04	Nd Nd
	4,6)-α-d-Gal*p-*(1→	98.9 5.00	69.3 3.85	71.4 3.91	70.7 4.18	72.6 4.19	65.0 3.93 a/3.67 b
	→2)-*α*-d-Rha*p-*(	92.1 5.07	77.7 3.86	69.3 3.68	72.6 3.35	69.0 3.93	16.5 1.17

Note: a = α, b = β.

**Table 7:** In vivo anti-diabetic experiment of ADPs60-F3.

Glucose Concentration (mg/dL)			
Groups	0 min	60 min	120 min
Control	85.35 ± 2.31	83.25 ± 1.83	84.06 ± 2.62
Model	85.24 ± 1.64 ^##^	246.36 ± 1.35 ^##^	252.18 ± 3.65 ^##^
Metformin	84.35 ± 2.51 ^##^	249.18 ± 3.62 ^##^	147.68 ± 2.53 ^##^
ADPs60-F3-L	83.62 ± 4.28 ^##^	261.32 ± 2.88 ^##,^**	208.47 ± 1.26 ^##,^**
ADPs60-F3-M	87.08 ± 2.39 ^##^	253.71 ± 2.58 ^##,^**	187.25 ± 2.38 ^##,^**
ADPs60-F3-H	84.75 ± 4.76 ^##^	243.86 ± 3.81 ^##,^**	186.39 ± 4.45 ^##,^**

All values represent the mean ± SEM (*n* = 6) for normal control, disease model, positive metformin control, and disease model with ADPs60-F3-L (100 mg/kg, ADPs60-F3), ADPs60-F3-M (300 mg/kg, ADPs6-F3), and ADPs60-F3-H (500 mg/kg, ADPs60-F3). ^##^ Significantly different vs. normal control at *p* < 0.01. ** Significantly different vs. model at *p* < 0.01.

## Data Availability

The original contributions presented in the study are included in the article, further inquiries can be directed to the corresponding author.
